# Epidemiological and clinical features of invasive pneumococcal disease caused by serotype 12F in adults, Japan

**DOI:** 10.1371/journal.pone.0212418

**Published:** 2019-02-21

**Authors:** Reiko Shimbashi, Bin Chang, Yoshinari Tanabe, Hiroaki Takeda, Hiroshi Watanabe, Tetsuya Kubota, Kei Kasahara, Kengo Oshima, Junichiro Nishi, Takaya Maruyama, Koji Kuronuma, Jiro Fujita, Tatsuki Ikuse, Yuki Kinjo, Motoi Suzuki, Anusak Kerdsin, Tomoe Shimada, Munehisa Fukusumi, Keiko Tanaka-Taya, Tamano Matsui, Tomimasa Sunagawa, Makoto Ohnishi, Kazunori Oishi, and the Adult IPD Study Group

**Affiliations:** 1 Field Epidemiology Training Program, National Institute of Infectious Diseases, Tokyo, Japan; 2 Division of Global Infectious Diseases, Department of Infection and Epidemiology, Graduate School of Medicine, Tohoku University, Sendai, Japan; 3 Infectious Diseases Surveillance Center, National Institute of Infectious Diseases, Tokyo, Japan; 4 Department of Bacteriology I, National Institute of Infectious Diseases, Tokyo, Japan; 5 Department of Respiratory Medicine, Niigata Prefectural Shibata Hospital, Shibata, Japan; 6 Department of Respiratory Medicine, Yamagata Saisei Hospital, Yamagata, Japan; 7 Department of Infection Control and Prevention, Kurume University School of Medicine, Kurume, Japan; 8 Department of Hematology and Respiratory Medicine, Kochi Medical School, Kochi University, Kochi, Japan; 9 Center for Infectious Diseases, Nara Medical University, Nara, Kashihara, Japan; 10 Department of Infection Control and Laboratory Diagnostics, Internal Medicine, Tohoku University Graduate School of Medicine, Miyagi, Sendai, Japan; 11 Department of Microbiology, Kagoshima University Graduate School of Medical and Dental Sciences, Kagoshima, Japan; 12 Department of Medicine, National Hospital Organization, Mie Hospital, Tsu, Japan; 13 Department of Respiratory Medicine and Allergology, Sapporo Medical University School of Medicine, Sapporo, Japan; 14 Department of Infectious, Respiratory and Digestive Medicine, Graduate School of Medicine, University of the Ryukyus, Nishihara, Japan; 15 Department of Pediatrics, Niigata University Graduate School of Medical and Dental Sciences, Niigata, Japan; 16 Department of Chemotherapy and Mycoses, National Institute of Infectious Diseases, Department of Bacteriology, Tokyo, Japan; 17 The Jikei University School of Medicine, Tokyo, Japan; 18 Department of Clinical Medicine, Institute of Tropical Medicine, Nagasaki University, Nagasaki, Japan; 19 Faculty of Public Health, Kasetsart University Chalermphrakiat Sakon Nakhon Province Campus, Sakon Nakhon, Thailand; Universidade de Lisboa Faculdade de Medicina, PORTUGAL

## Abstract

Enhanced surveillance of invasive pneumococcal disease (IPD) in adults was conducted during April 2013–March 2018 in 10 of 47 prefectures in Japan, and a total of 1277 IPD patients were enrolled. An emergence of IPD caused by serotype 12F was identified during May 2015–March 2018 through this surveillance. 12F isolates were composed of four related sequence types. In total, 120 patients with 12F IPD were reported during this period. To characterize the clinical features of 12F IPD, the disease characteristics of these patients were compared with those of 1157 patients with non-12F IPD. Compared with the non-12F IPD patients, a significantly lower proportion of 12F IPD patients was aged 65 years or older (55% vs. 70%), vaccinated with 23-valent pneumococcal polysaccharide (4% vs. 14%), had comorbid illness (65% vs. 77%), or were immunocompromised (19% vs. 30%; all *P* < 0.05). No significant difference in the proportion of case fatalities was found between the two groups. The proportions of those aged 65 years or older (53% vs. 69%) and with bacteremic pneumonia (35% vs. 69%) were significantly lower in 17 patients who died from 12F IPD than in 205 patients who died from non-12F IPD (all *P* < 0.05). Differences in clinical features were similarly found between 12F IPD patients and patients in low- or intermediate-level invasive potential serogroups. Our data demonstrated that serotype 12F was associated with IPD in younger adults and a lower proportion of comorbid illness, including immunocompromised conditions, in adult IPD, suggesting the high invasive potential of the serotype 12F. In addition, patients who died from 12F IPD were younger and had proportionately more bacteremia without focus. These findings may provide new insight into the pathogenesis of IPD in adults caused by 12F serotype with a high invasive potential.

## Introduction

S*treptococcus pneumoniae* (*S*. *pneumoniae*) is harbored in the human nasopharynx, and incidentally invades the bloodstream to cause invasive pneumococcal disease (IPD) [1]. The expression of a polysaccharide capsule, which includes more than 90 serotypes, contributes to the invasive property of this pathogen [2]. Previous studies demonstrated that invasive disease potential of different pneumococcal serotypes, and confirmed that capsular serotype is a major determinant of both the duration of carriage and attack rates in children [3–5]. Another study also indicated that the serotype appeared to be independently associated with IPD severity in adults [6].

The Japanese government decided to subsidize a 3+1 schedule of heptavalent pneumococcal conjugate vaccine (PCV7) for children less than 5 years of age in November 2010. PCV7 was included in the routine schedule in April 2013, and was replaced with a 13-valent pneumococcal conjugate vaccine (PCV13) in November 2013. Consequently, a high vaccination rate (94.4%) of a 3+1 schedule of PCV13 was observed in children at 23 months of age in 2016 [7]. For adults, a 23-valent pneumococcal polysaccharide vaccine (PPSV23) was licensed in 1988 and included in routine immunization in October 2014 of individuals aged 65 years or older. An interim program of routine immunization of PPSV23 is being conducted for five years until March 2019, of individuals aged 65 years, 70 years, 75 years, 80 years, 85 years, 90 years, 95 years, and 100 years or older. PCV13 was licensed for adults aged 65 years or older in 2014, and available on a voluntary basis.

*S*. *pneumoniae* serotype 12F is rarely found among colonizing isolates, but causes invasive disease in children and adults [6, 8]. Furthermore, serotype 12F is known to cause outbreaks in some institutions [9, 10] and community settings [11–16]. A high attack rate and a short duration of carriage, which were characteristics for serogroups of highly invasive disease potential, were confirmed in serotype 12F among children [5]. We recently identified an emergence of 12F IPD during May 2015–March 2018 via enhanced IPD surveillance of adults in 10 of 47 prefectures in Japan. The aim of this study was to characterize the epidemiological and clinical features of adult IPD caused by 12F serotype which is included in PPSV23 serotypes, but not in PCV13 serotypes.

## Materials and methods

### Adult IPD surveillance and case definition

A national surveillance program (National Epidemiological Surveillance of Infectious Diseases: NESID) for IPD has been implemented under the Infectious Diseases Control Law of Japan since April 2013. Physicians in all clinics and hospitals are required to notify all cases to the local public health authorities. Enhanced surveillance was implemented, with adult IPD cases reported to the NESID by the Adult IPD Study Group (http://www.nih.go.jp/niid/ja/ibi.html) in 10 prefectures (Hokkaido, Miyagi, Yamagata, Niigata, Mie, Nara, Kochi, Fukuoka, Kagoshima, and Okinawa) in Japan between April 2013 and March 2018. A case of IPD was defined as detection of *S*. *pneumoniae* by bacterial culture, and of *S*. *pneumoniae*-specific DNA by polymerase chain reaction (PCR) in normally sterile sites such as blood and/or cerebrospinal fluid (CSF); all IPD patients older than 15 years of age were enrolled in this study [17]. The sociodemographic and clinical data on age, sex, smoking history, alcohol abuse, hospitalization, intensive care unit (ICU) admission, PPSV23 vaccination within 5 years, preceding infection with influenza virus, comorbid illness, immunocompromised condition, asplenia/splenic hypoplasia (hyposplenia) or splenectomy, and outcome were collected using a case report form. This study was reviewed and approved by the Ethics Committee of NIID (no. 707) and conducted according to the principles expressed in the Declaration of Helsinki. Informed consent was waived because the data do not contain any patient identifiers and samples were taken as part of standard patient care.

### Bacteriological analysis

All pneumococcal isolates, including isolates serotyped by multiplex serotyping PCR at the prefectural public health institutes, were sent to the Department of Bacteriology I, National Institute of Infectious Diseases (NIID) and serotyped using a capsule Quellung reaction with rabbit antisera (Statens Serum Institute, Copenhagen, Denmark), as described elsewhere [18]. Multilocus sequence typing (MLST) of each isolate was performed for 12F isolates as previously described [19]. The resulting sequence types (STs) were assigned by using the *S*. *pneumoniae* MLST database [20]. eBURST was used to identify the clonal complexes for these *S*. *pneumoniae* serotype 12F isolates using retrieved MLST allelic profiles and STs from the *S*. *pneumoniae* MLST database. One isolate per patient was included in this analysis. Serotypes were classified into one of three groups: high invasive potential (serogroups 1, 5, and 7), intermediate invasive potential (serogroups 4, 9, 14, and 18), and low invasive potential (serogroups 3,6, 8, 15, 19, 23, and 33), according to the report by Jansen, et al. [6].

### Statistical analysis

The demographic data and clinical characteristics of 12F IPD and non-12F IPD cases were compared. Two-tailed P value was calculated by the Mann–Whitney U test for continuous variables. Odds ratios and confidence intervals were calculated using logistic regression model. All analyses were carried out using Stata software version 15 (StataCorp, College Station, Texas).

## Results

A total of 2544 cases of IPD were reported to the NESID from 10 prefectures between April 2013 and March 2018. Of these, 1296 IPD cases were enrolled into the study. 1292 of which were diagnosed by positive bacterial culture of sterile samples. Since 16 isolates were not available for serotyping, 1276 isolates were serotyped by Quellung reaction.

Four cases were diagnosed by positive PCR targeting *lytA*, and serotyped by multiplex serotyping PCR. Of 1280 cases in which the causative isolate was serotyped, three cases were excluded for study analysis because the information of sex was not available. Consequently, the infecting serotype was determined for a total of 1277 IPD cases.

### Serotype distribution

The serotype distribution of IPD isolates for these 1277 cases is shown in S1 Fig. While the proportions of all PCV7 serotypes, except for serotype 14 in 2013, in the IPD isolates were less than 4%, serotypes 3 and 19A were the leading serotypes in these IPD cases until 2015, after which they were replaced by serotype 12F during 2016–2017. Consequently, the proportion of non-PCV13 serotypes in the IPD isolates significantly increased in 2016 (69%) and 2017 (69%) compared with 2013–2015 (56% in 2013, 52% in 2014, 59% in 2015; *P*<0.0001). A total of 752 IPD isolates were classified into three serogroups: high invasive potential (n = 44), intermediate invasive potential (n = 39), and low invasive potential (n = 669) (S1 Table).

### 12F IPD and MLST analysis

**Fig 1:**
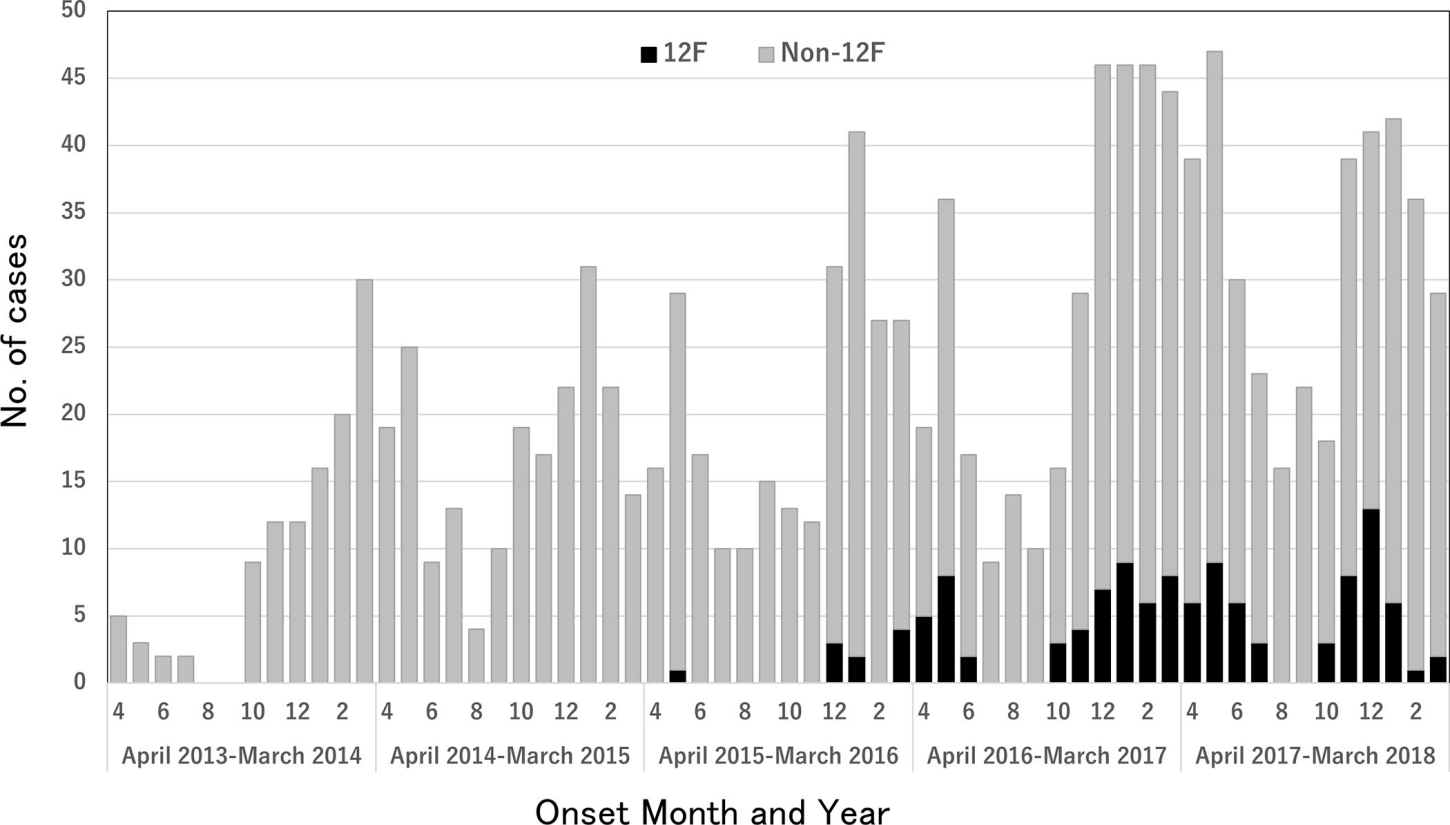
Epidemiological curve of invasive pneumococcal disease (IPD) among adults between April 2013 and March 2017 in 10 provinces, Japan. Closed bar denotes 12F serotype IPD, open bar denotes non-12F serotype IPD.

**Fig 2:**
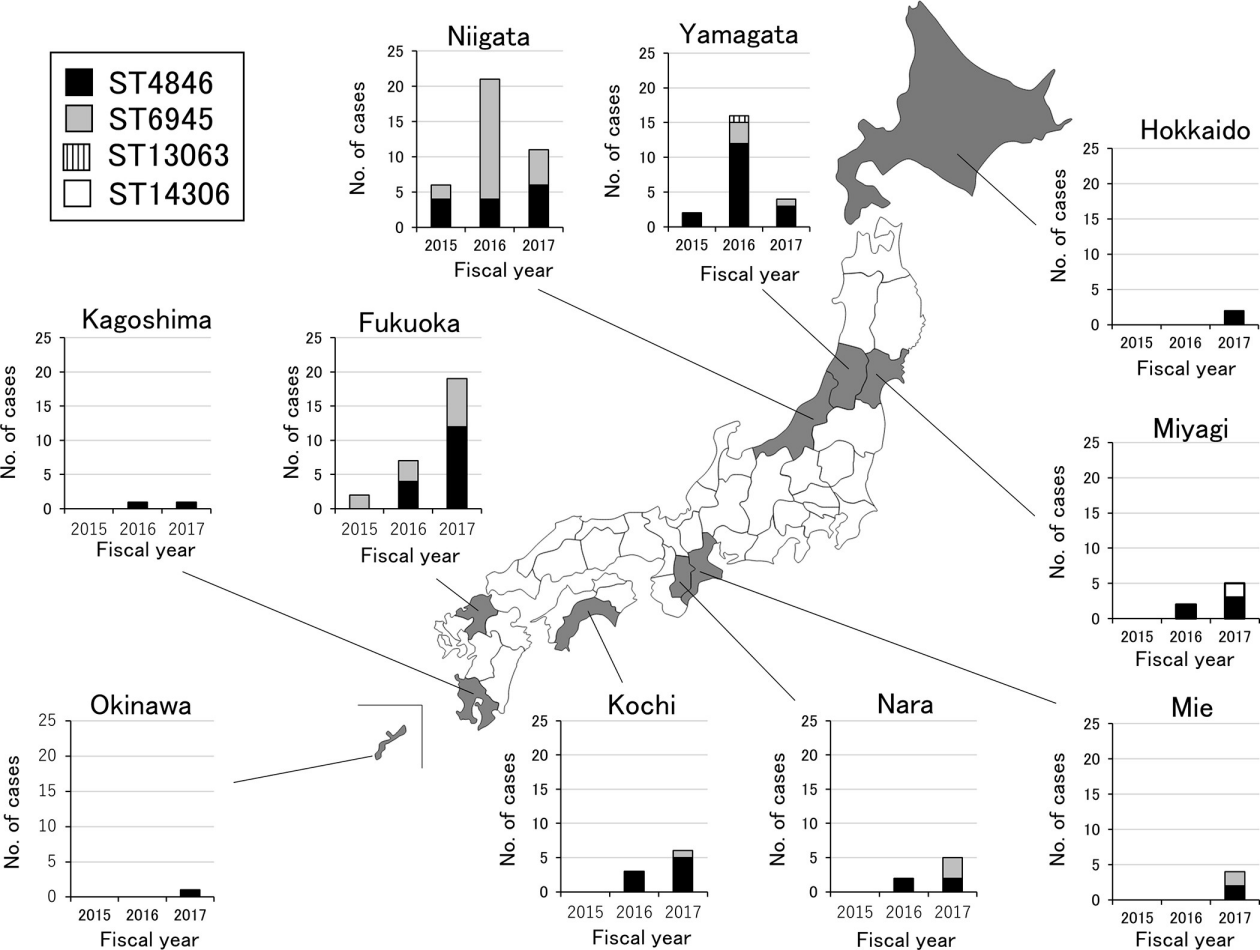
The number of cases of invasive pneumococcal disease in Japan caused by 12F serotype with sequence types 4846 (closed bar), 6945 (grey bar), 13063 (stripe bar) and a new sequence type (open bar) in 10 prefectures during April 2015 to March 2016, April 2016 to March 2017, and April 2017 and March 2018.

No case of serotype 12F IPD was reported between March 2013 and April 2015. However, after one case of 12F IPD with sequence type (ST) 4846 was identified in Niigata Prefecture in May 2015, one hundred and twenty cases were reported in all of ten prefectures until March 2018 (Figs 1 and 2). The majority of 12F IPD cases were reported from Niigata (n = 38), Fukuoka (n = 28), and Yamagata (n = 22) Prefectures. MLST analysis of a total of 120 isolates of serotype 12F demonstrated that they belonged to four STs: ST4846 (n = 71), ST6945 (n = 46), ST14306 (n = 2), and ST13063 (n = 1). A local outbreak of 12F IPD was transiently observed among children and adults in Tsuruoka city, Yamagata Prefecture, from March to May, 2016 [16]. No cluster of 12F IPD cases was identified in any institution in these areas, suggesting that disease transmission occurred in the community setting.

Japanese 12F isolates including ST4846, ST6945, ST13063, and ST14306 are clustered in clonal complex 1527 (S2 Fig). The ST13063 differs in one base of the *ddl* gene from ST4846, and ST6945 differs in two alleles (*recP* and *xpt*) from ST4846. The ST14306 differs from in one base of the *gdh* gene from ST4846, and is derived from ST4846. We also compared the MLST type with 12F isolates reported in the global PubMLST database. The STs 218, 989, 4846, and 6945 are the major isolates submitted to the global database.

### Characteristics of 12F IPD

We analyzed the clinical characteristics in 1277 IPD patients who were enrolled and serotyped between April 2013 and March 2018. We compared the clinical characteristics of 120 12F IPD patients with those of 1157 non-12F IPD patients (Table 1). The median age (interquartile range) of 12F IPD patients (67 years; age range, 59–75 years) was significantly lower than that of non-12F IPD patients (71 years; age range, 62–82 years; *P* = 0.001). The proportions of men (*P* = 0.021) and pneumonia (*P* = 0.042) were also slightly lower in 12F IPD than in non-12F IPD patients. In addition, the proportions of vaccination with PPSV23 (*P* = 0.013), comorbid illness (*P* = 0.003), immunocompromised conditions (*P* = 0.013), and patients aged 65 years or older (*P* = 0.001) were significantly lower in 12F IPD than in non-12F IPD patients. No significant difference was found between the two groups in the proportion of asplenia/hypoplasia or splenectomy, or fatalities.

**Table 1:** Comparison of clinical parameters in adult patients with IPD caused by 12F serotype and non-12F serotypes.

Total cases	All serotypes (n = 1277)	12F (n = 120)	Non-12F (n = 1157)	12F vs. Non-12F OR (95% CI)	*P*-value*
	n (%)				
Male	775 (61)	61 (51)	714 (62)	0.6 (0.4–0.9)	0.021
Smoking	412 (38)	33 (33)	379 (39)	0.8 (0.5–1.2)	0.295
Alcohol abuse	214 (20)	18 (19)	196 (20)	0.9 (0.5–1.6)	0.819
Hospitalization	1141 (95)	113 (99)	1028 (94)	7.1 (1.0–52.0)	0.052
ICU admission	325 (28)	36 (33)	289 (27)	1.4 (0.9–2.1)	0.159
Preceding influenza	56 (6)	2 (2)	54 (6)	0.3 (0.1–1.4)	0.131
Vaccination history of PPSV23	128 (13)	4 (4)	124 (14)	0.3 (0.1–0.8)	0.013
Comorbid illness	917 (76)	73 (65)	844 (77)	0.5 (0.4–0.8)	0.003
Immunocompromised condition	352 (29)	21 (19)	331 (30)	0.5 (0.3–0.9)	0.013
Asplenia/hyposplenia or splenectomy	53 (4)	4 (3)	49 (4)	0.8 (0.3–2.2)	0.633
Age group					
15-64y	406 (32)	54 (45)	352 (30)	Ref	
65y+	871 (68)	66 (55)	805 (70)	0.5 (0.4–0.8)	0.001
Clinical presentations					
Bacteremia	213 (17)	26 (22)	187 (16)	Ref	
Meningitis	191 (15)	15 (13)	176 (15)	0.6 (0.3–1.2)	0.151
Pneumonia	764 (60)	59 (49)	705 (61)	0.6 (0.4–1.0)	0.042
Others	109 (9)	20 (17)	89 (8)	1.6 (0.9–3.1)	0.139
Fatal outcome	222 (17)	17 (14)	205 (18)	0.8 (0.4–1.3)	0.330

*Logistic regression model. Others: arthritis, endocarditis, sinusitis, otitis media, vertebritis, cholecystitis, aortic aneurysm, pleurisy, and others.

To confirm the high invasive potential of the 12F serotype, in the present study we also compared the clinical characteristics of 12F IPD patients (n = 1277) to patients with IPD caused by serogroups with high, intermediate, or low invasive potential (S2 Table). The proportions of comorbid illness (*P* = 0.004), immunocompromised condition (*P* = 0.014), age older than 65 years or older (*P* = 0.001) and pneumonia (*P* = 0.026) were significantly lower in 12F IPD patients compared with patients with IPD caused by serogroup with low invasive potential (n = 669). We also found a difference in the frequency of comorbid illness (*P* = 0.013) and patients aged 65 years or older (*P* = 0.017) between 12F IPD patients and those with IPD caused by serogroup with intermediate invasive potential (n = 39), though no difference was found in the proportion of immunocompromised condition (*P* = 0.912), or pneumonia (*P* = 0.164). There was no difference between 12F IPD patients and those with IPD caused by high invasive potential (n = 44) in the proportion with comorbid illness (*P* = 0.214) or immunocompromised condition (*P* = 0.449), although there was a difference in the proportion of patients with pneumonia (*P* = 0.011).

The proportions of patients aged 65 years or older (*P* = 0.022; Table 2) and who had bacteremic pneumonia (*P* = 0.002) were significantly lower in the 17 fatal patients with 12F IPD than in 205 fatal patients with non-12F IPD. The proportion of asplenia/hyposplenia or splenectomy cases tended to be higher in patients with fatal 12F IPD than in patients with fatal non-12F IPD, although the difference was not significant.

**Table 2:** Comparison of clinical parameters in adult patients with fatal IPD caused by 12F serotype and non-12F serotypes.

	All serotypes (n = 222)	12F (n = 17)	Non-12F (n = 205)	12F vs Non-12F OR (95% CI)	*P*-value*
	n (%)				
Age group					
15-64y	52 (23)	8 (47)	44 (21)	Ref	
65y+	170 (77)	9 (53)	161 (79)	0.3 (0.1–0.8)	0.022
Asplenia/hyposplenia or splenectomy	14 (6)	3 (18)	11 (5)	3.7 (0.9–15.0)	0.062
Clinical presentations					
Bacteremia	47 (21)	9 (53)	38 (19)	Ref	
Meningitis	18 (8)	2 (12)	16 (8)	0.5 (0.1–2.7)	0.445
Pneumonia	148 (67)	6 (35)	142 (69)	0.2 (0.1–0.5)	0.002
Others	9 (4)	0 (0)	9 (4)	NA	NA

*Logistic regression model. Others: arthritis, endocarditis, sinusitis, otitis media, vertebritis, cholecystitis, aortic aneurysm, pleurisy, and others. NA: not applicable.

The clinical characteristics of patients with fatal 12F IPD (n = 17) were also compared with patients with fatal IPD patients caused by serogroup with low invasive potential (n = 136) (S3 Table). The proportions of patients aged 65 years or older (*P* = 0.03) and with pneumonia (*P* = 0.001) were significantly lower in the fatal 12F IPD than in fatal IPD caused by serogroup with low invasive potential. The proportion of asplenia/hyposplenia or splenectomy cases was slightly higher in patients with fatal 12F IPD than in patients with fatal IPD caused by serogroup with low invasive potential (*P* = 0.046).

## Discussion

In the present study, we identified an emergence of 12F IPD among adults in Japan during 2015–2017. Our descriptive analyses demonstrated significantly lower proportions of comorbid illness, immunocompromised conditions, PPSV23 vaccination, and age 65 years or older in 12F IPD patients compared with non-12F IPD patients. We also found that patients who died from 12F IPD were younger and more frequently had associated bacteremia.

Although most of the 12F IPD cases (73%) in the present study were reported in three prefectures, the transmission linkages between the cases remained unclear. A recent report from Israel demonstrated a sharp increase, long duration and predominance of 12F IPD after sequential introduction of PCV7 and PCV13 for children [21]. The majority of 12F serotypes were isolated from IPD patients of <5 years. The authors stated that emergence of 12F IPD might be attributable to serotype replacement after PCV introduction. Furthermore, a recent report from the United Kingdom also demonstrated a rapid increase of incidence of IPD caused by serotypes 12F, 8, and 9N, primarily in adults >65 years since 2012 (3 years after introduction of PCV13) [22]. A continuous increase in IPD incidence caused by these replacement serotypes was observed among children and adults until 2017.

A transient predominance of the 12F serotype was reported as the leading cause of adult IPD from 2006 to 2007 before introduction of PCV in Japan [23]. However, the reasons for the emergence of the 12F IPD during 2006–2007 remain uncertain. While a gradual increase in the proportion of non-PCV13 serotypes was observed in the adult IPD isolates following the introduction of PCV7/PCV13 in 2010 [24,25], the 12F IPD suddenly reemerged from 2015 and remained at high proportions in a total number of IPD isolates in 2016 (16%) and 2017 (15%) in this study (S1 Fig). This proportion slightly decreased to 12% during April–November 2018 (data not shown). Therefore, it is uncertain whether the current emergence of 12F IPD shown in Japan is attributable to serotype replacement after PCV introduction or to a transient epidemic. 12F isolates identified during the current epidemic belonged to four STs—4846, 6845, 13063 and 14306—that were distinct from ST220, which was detected in a previous epidemic of 12F IPD among adults in Japan [26].

The significantly lower median age and proportion of comorbid illness in 12F IPD patients observed in the present study is in agreement with a report of an outbreak of 12F IPD in Canada [14]. The authors reported that 12F IPD patients were more likely than non-12F IPD patients to be younger adults aged 18–54 years than non-12F IPD patients, and the proportion of preexisting health conditions in 12F IPD patients was lower than that in non-12F IPD patients. Jansen et al. reported that highly invasive serotypes were often associated with milder disease manifestation and more often affected individuals without comorbidities [6]. They demonstrated that the proportions of patients older than 79 years (12.4%), with any comorbidity (56.1%), and with immunocompromised conditions (9.9%) were significantly lower in patients with IPD caused by the serogroup with high invasive potential than in patients with IPD caused by the serogroup with intermediate or low invasive potential. Collectively, data from previous studies [5, 6, 14] and our present data may suggest that serotype 12F is a serogroup with high invasive potential because adult patients with 12F IPD have the characteristic clinical features of younger age and lower proportions of comorbid illness and immunocompromised conditions. Furthermore, our comparisons between 12F IPD patients and patients with IPD caused by the serogroup with low or intermediate invasive potential supports the notion that serotype 12F is associated with a high invasive potential. The clinical similarity between 12F IPD and IPD caused by the serogroups with high invasive potential also suggests an association between high invasive potential and serotype 12F. Although we found higher proportions of asplenia/hyposplenia or splenectomy cases in cases with fatal 12F IPD than in fatal non-12F IPD or fatal IPD caused by the serogroup with low invasive potential, it remains uncertain whether adults underlined by asplenia/hyposplenia or splenectomy have an increased risk of death from 12F IPD.

No difference was found in the case fatality rates between 12F and non-12F IPD patients in this study, which is consistent with a previous report [14]. Although Jansen, et al. demonstrated that the serogroup of low invasive potential was associated with increased case fatality rates, compared with the serogroup of high invasive potential [6], we found no difference in the case fatality rates between the serogroups of high, intermediate, and low invasive potential (S2 Table). Further studies are required to clarify the differences in the case fatality rates among the invasive potential serogroups.

The average proportion of PPSV23 vaccination in the target groups of individuals aged 65 years or over from April 2015 to March 2016 has been reported to be 40.8% [27]. By contrast, the proportion of patients vaccinated with PPSV23 in the total cohort of IPD cases in the present study was only 13%, while it was 18% for patients aged older than 65 years. A significantly lower proportion (4%) of 12F IPD patients in this study who had received PPSV23 containing 12F polysaccharide antigen may suggest that they were selected from the adult population who had not been recently vaccinated, because the effectiveness of this vaccine against vaccine-type IPD in adults has been established [28,29]. This possibility is supported by previous reports on the vaccine effectiveness of PPSV23 against 12F IPD in younger adults and in the elderly in England and Wales [30, 31]. Therefore, it is strongly recommended for unvaccinated adults aged 65 years or older, who are a target of routine immunization, to receive PPSV23 vaccination.

This study has several limitations. First, the proportion of cases available for both case report data and isolate data was 50.9% (1296/2544) of the cases reported to national surveillance sites from the 10 selected prefectures in this study, and reporting of some variables was incomplete. Therefore, the results may not be representative of the population of interest. Second, the number of registered cases in the enhanced surveillance data increased annually, which suggests the possibility of missing cases of 12F IPD during the study period. Third, the analysis on the disease characteristics in this study may be insufficient because of a small number of 12F IPD case and fatal 12F IPD case.

In conclusion, we demonstrated that 12F IPD may occur in younger adults without comorbid illness, including immunocompromised conditions. The clinical features of 12F IPD may suggest a high invasive potential of the serotype 12F. 12F IPD may cause death at a younger age, and might be more likely to be associated with bacteremia without focus. Therefore, further clinical and basic studies are required to clarify the mechanism of the pathogenesis of 12F IPD in adults. In addition, the prevalence of 12F IPD in Japan should be carefully monitored through enhanced surveillance of IPD.

## Supporting information

S1 Fig Distribution of the serotypes of causative pneumococcal isolates from 1277 adult patients with invasive pneumococcal disease from 10 prefectures in Japan, from April 2013 to March 2018. The vertical axis indicates the proportion of individual serotypes in the total number of isolates for each year. 6A and 11E are not included in 23-valent pneumococcal polysaccharide vaccine. Abbreviations: PCV7, heptavalent pneumococcal conjugate vaccine; PCV13, 13-valent pneumococcal conjugate vaccine; PPSV23, 23-valent pneumococcal polysaccharide vaccine.
(TIF)

S2 Fig An eBURST Analysis of *Streptococcus pneumoniae* Serotype 12F Isolates Globally and from Japan. The 12F isolates with sequence types (ST) 4846, 4945, 13063, and 14308 are indicated by red circles. The other 12F isolates in Japan with STs 220, 4845, 11186, and 12003, not included in our study, are shown in orange. The predicted founders of STs are located in the center of the clusters and indicated by blue circles. The size of the circles is relative to the number of isolates with respective ST present in the database. Two isolates with the new ST are under submission for *Streptococcus pneumoniae* MLST website.
(JPG)

S1 Table Serogroups of IPD isolates. (DOCX)

S2 Table Comparison of clinical parameters in adult patients with IPD caused by 12F serotype and IPD caused by serogroups of high, intermediate and low invasive potential. (PDF)

S3 Table Comparison of clinical parameters in adult patients with fatal IPD caused by 12F serotype and fatal IPD caused by high, intermediate and low serogroups of invasive potential. (PDF)
