# Supplementary figures and images for: Epidemiological and clinical features of invasive pneumococcal disease caused by serotype 12F in adults, Japan
Source: PLoS One. 2019 Feb 21;14(2):e0212418. doi: 10.1371/journal.pone.0212418 (PMC6383924; doi:10.1371/journal.pone.0212418)

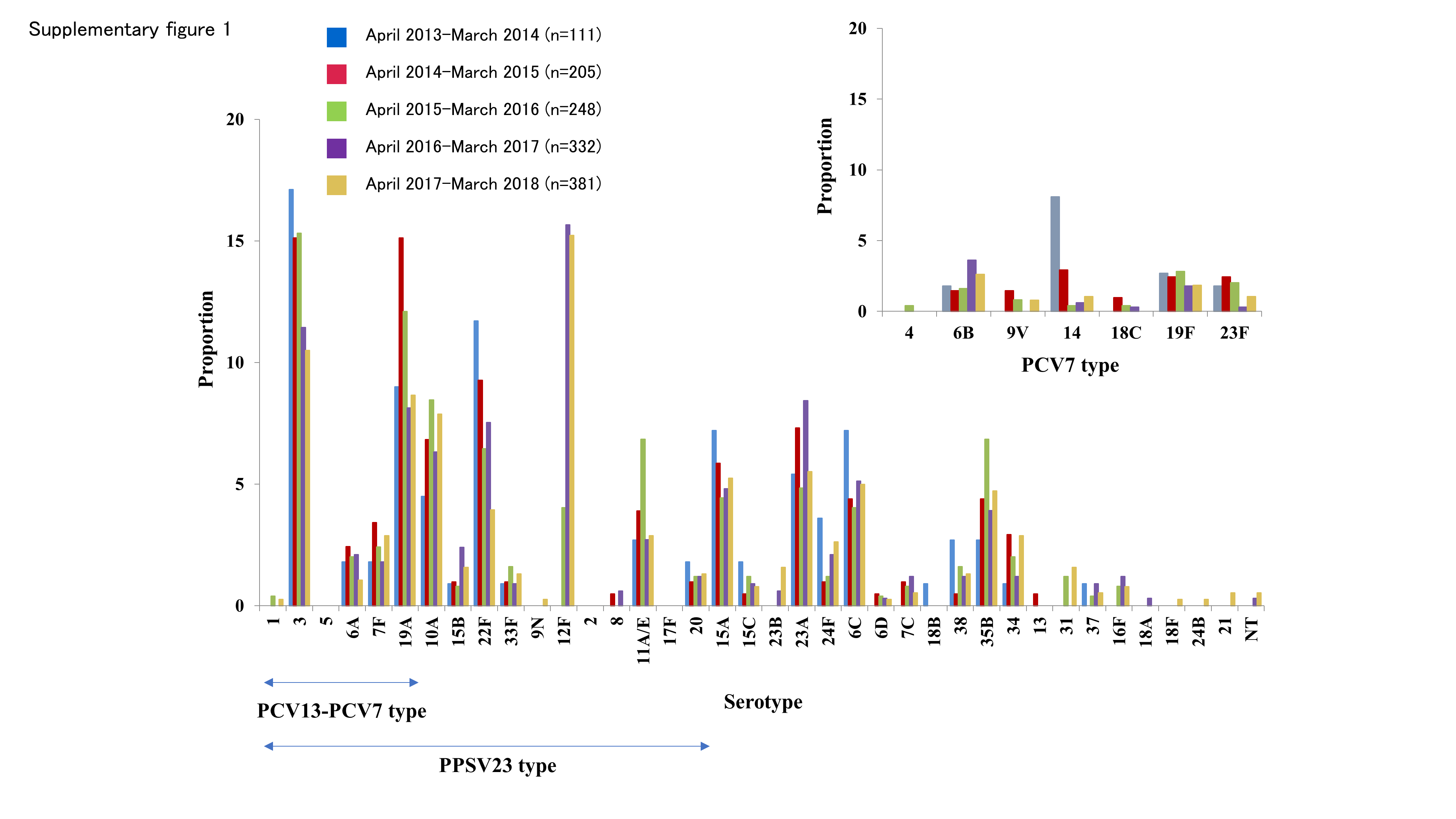

Supplement: S1 Fig — The vertical axis indicates the proportion of individual serotypes in the total number of isolates for each year. 6A and 11E are not included in 23-valent pneumococcal polysaccharide vaccine. Abbreviations: PCV7, heptavalent pneumococcal conjugate vaccine; PCV13, 13-valent pneumococcal conjugate vaccine; PPSV23, 23-valent pneumococcal polysaccharide vaccine. (TIF) [file pone.0212418.s001.tif]

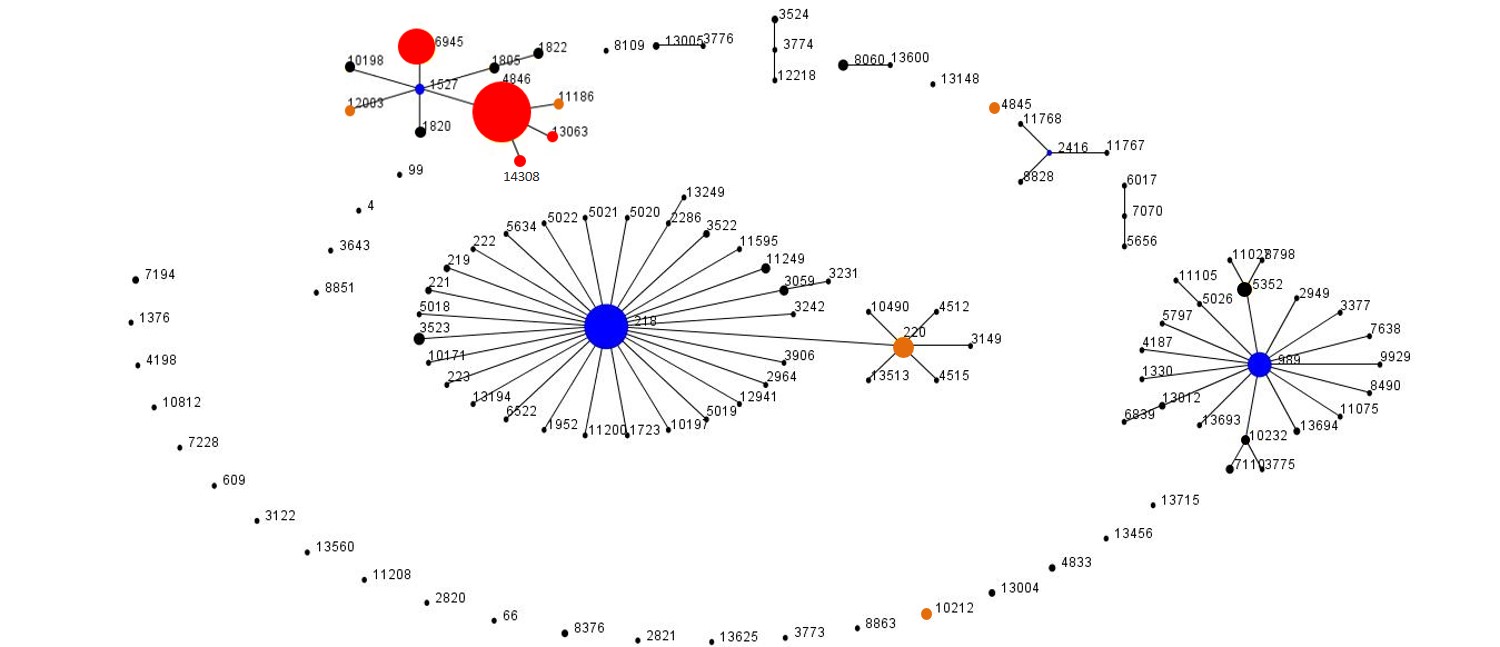

Supplement: S2 Fig — The 12F isolates with sequence types (ST) 4846, 4945, 13063, and 14308 are indicated by red circles. The other 12F isolates in Japan with STs 220, 4845, 11186, and 12003, not included in our study, are shown in orange. The predicted founders of STs are located in the center of the clusters and indicated by blue circles. The size of the circles is relative to the number of isolates with respective ST present in the database. Two isolates with the new ST are under submission for Streptococcus pneumoniae MLST website. (JPG) [file pone.0212418.s002.jpg]
