# Supplementary material for: Epidemiological and clinical features of invasive pneumococcal disease caused by serotype 12F in adults, Japan
Source: PLoS One. 2019 Feb 21;14(2):e0212418. doi: 10.1371/journal.pone.0212418 (PMC6383924; doi:10.1371/journal.pone.0212418)
Supplement: S1 Table — (DOCX) [file pone.0212418.s003.docx]

| Invasive disease potential | Serotypes | No. of cases | Total no. of cases by serogroups | |
| --- | --- | --- | --- | --- |
| High | 1 | 2 | | 44 |
|  | 5 | 0 | |  |
|  | 7C | 10 | |  |
|  | 7F | 32 | |  |
| Intermediate | 4 | 1 | | 39 |
|  | 9N | 1 | |  |
|  | 9V | 8 | |  |
|  | 14 | 22 | |  |
|  | 18A | 1 | |  |
|  | 18B | 1 | |  |
|  | 18C | 4 | |  |
|  | 18F | 1 | |  |
| Low | 3 | 166 | | 669 |
|  | 6A | 23 | |  |
|  | 6B | 31 | |  |
|  | 6C | 63 | |  |
|  | 6D | 4 | |  |
|  | 8 | 3 | |  |
|  | 15A | 67 | |  |
|  | 15B | 19 | |  |
|  | 15C | 12 | |  |
|  | 19A | 131 | |  |
|  | 19F | 28 | |  |
|  | 23A | 82 | |  |
|  | 23B | 8 | |  |
|  | 23F | 17 | |  |
|  | 33F | 15 | |  |
