# Supplementary material for: Epidemiological and clinical features of invasive pneumococcal disease caused by serotype 12F in adults, Japan
Source: PLoS One. 2019 Feb 21;14(2):e0212418. doi: 10.1371/journal.pone.0212418 (PMC6383924; doi:10.1371/journal.pone.0212418)
Supplement: S2 Table — (PDF) [file pone.0212418.s004.pdf]

Total cases	All serotypes (n=1277)	12 F serotype (n=120)	Invasive disease potential of serogreoups			12F vs. High	*P*-value*	12F vs. Intermediate	*P*-value*	12F vs. Low	*P*-value*
			High (n=44)	Intermediate (n=39)	Low (n=669)						
	n(%)										
Comorbid illness	917 (76)	73 (65)	33 (75)	33 (87)	486 (77)	0.6 (0.3-1.3)	0.214	0.3 (0.1-0.8)	0.013	0.5 (0.3-0.8)	0.004
Immunocompromised condition	352 (29)	21 (19)	6 (14)	7 (18)	191 (30)	1.5 (0.5-3.9)	0.449	1.1 (0.4-2.7)	0.912	0.5 (0.3-0.9)	0.014
Asplenia/ hyposplenia or splenectomy	53 (4)	4 (3)	1 (2)	1 (3)	25 (4)	1.5 (0.2-13.6)	0.728	1.3 (0.1-12.0)	0.812	0.9 (0.3-2.6)	0.824
Age group											
15-64y	406 (32)	54 (45)	13 (30)	9 (23)	201 (30)	Ref		Ref		Ref	
65y+	871 (68)	66 (55)	31 (70)	30 (77)	468 (70)	0.5 (0.2-1.1)	0.077	0.4 (0.2-0.8)	0.017	0.5 (0.4-0.8)	0.001
Clinical presentations											
Bacteremia	213 (17)	26 (22)	3 (7)	7 (18)	105 (16)	Ref		Ref		Ref	
Meningitis	191 (15)	15 (13)	4 (9)	0 (0)	95 (14)	0.4 (0.1-2.2)	0.313	NA	NA	0.6 (0.3-1.3)	0.203
Pneumonia	764 (60)	59 (49)	35 (80)	31 (79)	425 (64)	0.2 (0.1-0.7)	0.011	0.5 (0.2-1.3)	0.164	0.6 (0.3-0.9)	0.026
Others	109 (9)	20 (17)	2 (5)	1 (3)	44 (7)	1.2 (0.2-7.6)	0.882	5.4 (0.6-47.4)	0.129	1.8 (0.9-3.6)	0.08
Fatal outcome	222 (17)	17 (14)	7 (16)	10 (26)	136 (20)	0.9 (0.3-2.3)	0.78	0.5 (0.2-1.2)	0.102	1.0 (0.9-1.0)	0.118

* Logistic regression model. Others: arthritis, endocarditis, sinusitis, otitis media, vertebritis, cholecystitis, aortic anuerism, pleusy, and others. NA: not applicable.
