# Supplementary material for: Epidemiological and clinical features of invasive pneumococcal disease caused by serotype 12F in adults, Japan
Source: PLoS One. 2019 Feb 21;14(2):e0212418. doi: 10.1371/journal.pone.0212418 (PMC6383924; doi:10.1371/journal.pone.0212418)
Supplement: S3 Table — (PDF) [file pone.0212418.s005.pdf]

Total cases	All serotypes (n=222)	12 F serotype (n=17)	Invasive disease potential of serogreoups			12F vs. Low	*P*−value*
			High (n=7)	Intermediate (n=10)	Low (n=136)		
	n(%)						
Comorbid illness	173 (81)	10 (63)	6 (86)	10 (100)	104 (80)	0.4 (0.1-1.3)	0.119
Iimmunocompromised condition	65 (30)	5 (33)	1 (14)	2 (20)	34 (26)	1.4 (0.5-4.5)	0.531
Aasplenia/ hyposplenia or splenectomy	14 (6)	3 (18)	1 (14)	0 (0)	6 (4)	4.6 (1.0-20.3)	0.046
Age group							
15−64y	52 (23)	8 (47)	1 (14)	1 (10)	30 (22)	Ref	
65y+	170 (77)	9 (53)	6 (86)	9 (90)	106 (78)	0.3 (0.1-0.9)	0.030
Clinical presentations							
Bacteremia	47 (21)	9 (53)	1 (14)	3 (30)	22 (16)	Ref	
Meningitis	18 (8)	2 (12)	2 (29)	0 (0)	9 (6)	0.5 (0.1-3.0)	0.486
Pneumonia	148 (67)	6 (35)	4 (57)	7 (70)	99 (73)	0.1 (0.0-0.5)	0.001
Others	9 (4)	0 (0)	0 (0)	0 (0)	6 (4)	NA	NA

* Logistic regression model. Others: arthritis, endocarditis, sinusitis, otitis media, vertebritis, cholecystitis, aortic anuerism, pleusy, and others. NA: not applicable.
